# The role of protein phosphorylation modifications mediated by iron metabolism regulatory networks in the pathogenesis of Alzheimer’s disease

**DOI:** 10.3389/fnagi.2025.1540019

**Published:** 2025-02-25

**Authors:** Fei-Xiang Liu, Shun-Zhi Yang, Kai-Kai Shi, Ding-Ming Li, Jia-bin Song, Lu Sun, Xue Dang, Jin-Yao Li, Zi-qi Deng, Min Zhao, Yan-Chen Feng

**Affiliations:** ^1^Department of Neuropsychiatry and Psychology, The First Affiliated Hospital of Henan University of Chinese Medicine, Zhengzhou, China; ^2^Hospital of Encephalopathy, The First Affiliated Hospital of Henan University of Chinese Medicine, Zhengzhou, China; ^3^The First Clinical Medical School, Henan University of Chinese Medicine, Zhengzhou, China; ^4^School of Medicine, Henan University of Chinese Medicine, Zhengzhou, China; ^5^College of Acupuncture, Moxibustion and Tuina, Henan University of Chinese Medicine, Zhengzhou, China; ^6^Traditional Chinese Medicine (Zhong Jing) School, Henan University of Chinese Medicine, Zhengzhou, China; ^7^School of Pharmacy, Henan University of Chinese Medicine, Zhengzhou, China

**Keywords:** iron, iron metabolism, protein phosphorylation, Alzheimer’s disease, post-translational modification of proteins

## Abstract

Alzheimer’s disease (AD) is a severe neurodegenerative disease characterized mainly by the formation of amyloid beta (Aβ) plaques and abnormal phosphorylation of tau. In recent years, an imbalance in iron homeostasis has been recognized to play a key role in the pathological process of AD. Abnormal iron accumulation can activate various kinases such as glycogen synthase kinase-3β, cyclin-dependent kinase 5, and mitogen-activated protein kinase, leading to abnormal phosphorylation of tau and amyloid precursor protein, and accelerating the formation of Aβ plaques and neurofibrillary tangles. In addition, iron-mediated oxidative stress not only triggers neuronal damage, but also exacerbates neuronal dysfunction by altering the phosphorylation of N-methyl-D-aspartate receptors and γ-aminobutyric acid type A receptors. Iron accumulation also affects the phosphorylation status of tyrosine hydroxylase, the rate-limiting enzyme for dopamine synthesis, interfering with the dopamine signaling pathway. On the other hand, iron affects iron transport and metabolism in the brain by regulating the phosphorylation of transferrin, further disrupting iron homeostasis. Therapeutic strategies targeting iron metabolism show promise by reducing iron accumulation, inhibiting oxidative stress, and reducing abnormal phosphorylation of key proteins. This article reviews the molecular mechanisms of phosphorylation modifications mediated by iron homeostasis imbalance in AD, and discusses the potential of interventions that regulate iron metabolism and related signaling pathways, providing a new theoretical basis for the treatment of AD.

## 1 Introduction

Alzheimer’s disease (AD) is the most common neurodegenerative disease in the elderly, caused by multiple factors such as genetics, the environment, and aging. The primary features of the disease include the formation of amyloid beta (Aβ) protein plaques and the over phosphorylation and aggregation of tau, ultimately leading to neurofibrillary tangles (NFTs) (Golde and Levey, 2023). The clinical manifestations of AD include memory loss, impaired language skills, decreased executive fßunction, and changes in behavior and mood. These symptoms gradually worsen, eventually leading to a complete loss of self-care capacity, placing a heavy burden on families and society (Kepp et al., 2023). Currently, more than 50 million people worldwide are affected by AD, and it is estimated that by 2050, this number will exceed 130 million (Ono et al., 2020). AD poses a major challenge to global public health. It is projected that by 2030, global medical and social care costs related to AD will reach 2 trillion United States dollars. These costs include direct medical expenses, long-term care costs, and the financial burden borne by unpaid caregivers (Frankish and Horton, 2017). These costs are expected to continue rising as the number of AD patients increases. Despite decades of research, scientists now have some understanding of the complex interactions between genetic and environmental risk factors in AD, but the only commonly used therapeutic drugs for specific symptoms of AD are four cholinesterase inhibitors and one N-methyl-D-aspartic acid receptor (NMDAR) inhibitor, and these drugs can only provide temporary relief (Shega et al., 2009). Therefore, due to the highly complex pathogenesis of AD and our continuously evolving understanding of it, there is currently no global cure, and finding effective prevention and treatment methods remains a major challenge for the scientific community.

As a core driver of neurodegenerative diseases, aging contributes to the pathological deposition of Aβ in 10–40% of cognitively normal elderly individuals without clinical symptoms. This suggests that simple Aβ deposition alone may not be sufficient to trigger the pathological cascade of AD (Vipin et al., 2019; Pelkmans et al., 2020). However, brain iron accumulation, which is not merely a physiological consequence of aging, may interact with Aβ deposition and act as a critical pathological factor in the development of AD. Neuropathological studies have further revealed that amyloid plaques and neurofibrillary tangles in the brain tissue of AD patients exhibit notable iron enrichment. Synchrotron radiation X-ray microscopic analysis confirmed the abnormal deposition of Fe^2 +^ in the core region of these plaques, with this excessive iron accumulation exacerbating Aβ aggregation and accelerating the progression of pathological responses (Jiang et al., 2009; Everett et al., 2014). Additionally, a large-scale study (*n* = 150) of cognitively normal individuals demonstrated that brain iron content was significantly negatively correlated with core cognitive functions, including episodic memory and visuospatial abilities, whereas the correlation between Aβ load and cognitive test scores was not statistically significant (Hedden et al., 2013; Chen et al., 2021). Another meta-analysis corroborated that although the overall cognitive Z-score of Aβ-positive individuals decreased by only 0.12, frontal iron deposition significantly accelerated cognitive decline, with its impact on cognitive deterioration surpassing that of Aβ deposition. Specifically, iron deposition increased the rate of cognitive decline by a factor of 2.3 (Hedden et al., 2013). Collectively, these findings suggest that brain iron accumulation not only contributes to cognitive impairment before Aβ deposition but also that its interaction with Aβ plays a more central role in the pathological mechanisms underlying AD.

The role of iron homeostasis in AD is a widely studied research area (Moos, 2002). Iron is an essential trace element in the human body, involved in various physiological functions such as oxygen transport, DNA synthesis, and energy metabolism (Lane et al., 2018). In the brain, iron is mainly found in neurons, glial cells, and a small amount in blood vessels (Connor and Menzies, 1995; McCarthy and Kosman, 2015). Iron in the blood normally binds to transferrin (Tf) to form the transferrin-iron complex (Tf-Fe), which then crosses the blood-brain barrier (BBB) via transferrin receptor (TfR)-mediated endocytosis. TfR1 on the surface of endothelial cells in the BBB specifically binds to the Tf-Fe complex, forming a receptor-ligand complex that enters the cell. In an acidic environment, iron is released from Tf and reduced from Fe^3 +^ to Fe^2 +^ by iron reductase (Mezzanotte and Stanga, 2024). Subsequently, Fe^2 +^ is transported intracellularly via iron transporters (e.g., divalent metal transporter 1, DMT1) and can be selectively released into neurons or glial cells, while unutilized Tf is recycled into circulation to maintain iron homeostasis (Ponka and Lok, 1999). The process of iron crossing the BBB is regulated by multilayered mechanisms, including iron regulatory proteins (IRPs) and hepcidin. IRPs regulate the expression of TfR and ferroportin by binding to iron-responsive elements (IREs) to meet cellular iron needs, while hepcidin controls the absorption and distribution of iron throughout the body by inhibiting ferroportin, ensuring iron homeostasis (Garza et al., 2020). Once the BBB is damaged by aging and/or high levels of oxidative stress, the random transport of Fe3^+^ and Fe2^+^ across the BBB leads to improper compartmentalization of ions in the brain (Bradbury, 1997). This complex system ensures precise iron delivery while preventing oxidative damage due to excessive accumulation.

In neurons, iron enters cells via TfR-mediated endocytosis and is stored in ferritin, where it plays a key role in storage and detoxification (Gammella et al., 2021). At the same time, Iron is also a necessary cofactor for the synthesis of various neurotransmitters and is essential for energy metabolism processes such as the tricarboxylic acid cycle and oxidative phosphorylation. It is critical for efficient nerve signal transmission, synapse formation, and overall neuronal function (Gammella et al., 2017; Geng et al., 2023). In addition, iron is a key element in the formation of antioxidant enzymes that protect nerve cells from damage caused by free radicals (Chen et al., 2023). Iron also plays an important role in the regeneration and repair of cells after nervous system damage, especially during myelination, when iron is essential for the formation of myelin sheaths (Santiago González et al., 2019). Maintaining appropriate iron levels is therefore essential for nervous system health, and imbalances in iron metabolism can negatively affect the normal development and function of the nervous system. Previous studies have shown (Raj et al., 2021) hat long-term exposure to heavy metals such as aluminum and iron can cause neurotoxicity and AD-like symptoms by inducing the accumulation of amyloid plaques, tau phosphorylation, increased oxidative stress, neuroinflammation, and cholinergic neuron degeneration in the brain. This has been further confirmed in animal experiments (Raj et al., 2021), in which AD-like symptoms were significantly induced in rats by administering aluminum chloride and iron by gavage, causing an increase in Aβ levels, tau phosphorylation, and nuclear factor kappa-B (NF-κB) pathway activation. Long-term inhalation of iron-rich combustion and friction-derived nanoparticles can also lead to the massive accumulation of these particles in the nuclei of neurons, glial cells, and endothelial cells in frontal white matter samples from urban residents. This impairs brain chromatin silencing and reduces DNA integrity, thereby increasing the risk of AD in young residents (Calderón-Garcidueñas et al., 2020). Therefore, abnormal iron accumulation in the brains of AD patients is closely linked to oxidative damage responses, neuronal apoptosis, ferritin dysregulation, and the expression of iron-related genes.

Protein phosphorylation plays a crucial role in the pathogenesis of AD (Ma et al., 2021). Phosphorylation is a key post-translational modification that regulates protein activity, stability, and interactions by adding phosphate groups to proteins (Vogel and Isono, 2020). Iron accumulation and abnormal phosphorylation of multiple proteins affect normal physiological functions, leading to neuronal damage and cognitive decline (Wang et al., 2023; Rajendran and Krishnan, 2024). Among these, abnormal phosphorylation of tau and amyloid precursor protein (APP) is one of the main pathological features of AD (Chen and Yu, 2023). Hyperphosphorylation of tau causes it to dissociate from microtubules, forming NFTs and disrupting neuronal structure and function (Lin et al., 2024). The phosphorylation of APP alters its metabolic pathway, promoting Aβ production and deposition to form amyloid plaques (Hajdú et al., 2023). Abnormal iron accumulation in the brain produces large amounts of reactive oxygen species (ROS) through the Fenton reaction, further promoting the phosphorylation of tau and APP, leading to neuronal damage and cognitive decline (Smith et al., 1997; Du et al., 2022). In this process, various kinases such as glycogen synthase kinase-3β (GSK3β), cyclin-dependent kinase 5 (CDK-5), and mitogen-activated protein kinase (MAPK) are activated, enhancing the phosphorylation of tau and APP, exacerbating pathological changes (Takashima et al., 1998; Kanno et al., 2016). In contrast, the use of iron chelating agents can inhibit the activity of these kinases, thereby reducing the phosphorylation of tau and APP, demonstrating the potential to slow the progression of AD (Guo et al., 2013a). Therefore, in-depth research on iron-mediated protein phosphorylation and the elucidation of its mechanism of action in AD will not only help reveal the pathological processes of AD, but also provide an important theoretical basis for developing new treatment strategies.

## 2 Current evidence of brain iron accumulation in different regions and its association with AD symptoms

A large number of studies have shown that the overload of brain iron content and iron deposits not only exacerbates cognitive dysfunction in AD animal models, but also serves as a biomarker of cognitive and age-related decline in elderly subjects, which is closely related to the lifelong stability of their intelligence (Penke et al., 2012; Schröder et al., 2013; Howard et al., 2022). Simultaneously, there are differences in iron demand and metabolic regulation mechanisms across different brain regions, and iron alterations are influenced by factors such as neuronal activity, blood-brain barrier function, oxidative stress, and protein deposition. Understanding these regional iron changes not only provides clinical value for the early diagnosis of AD but also enhances our understanding of the role of iron in AD pathology (Ward et al., 2014).

**FIGURE 1:**
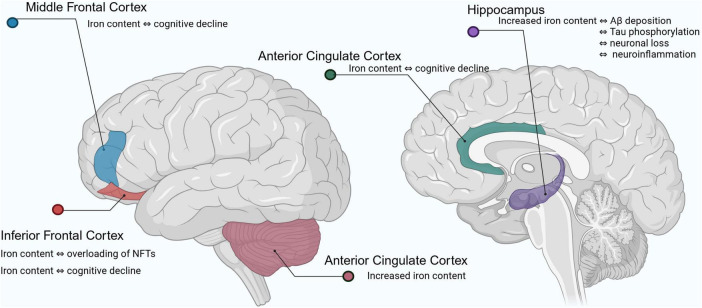
Changes in iron levels in the brain of Alzheimer’s patients. Aβ, amyloid beta; NFTs, neurofibrillary tangles.

In recent years, MRI has been used to study the relationship between brain iron levels and cognitive parameters in living individuals. Numerous studies on the quantitative analysis of brain iron content in human subjects with AD have shown that brain iron accumulation primarily affects the following areas (Ayton et al., 2020; Ayton et al., 2021): iron content in the inferior temporal cortex is significantly associated with NFTs overload and strongly correlates with cognitive decline; iron loading in the middle frontal cortex is associated with cognitive decline despite no significant increase in total iron levels; iron loading in the anterior cingulate cortex is linked to cognitive decline; iron content in cerebellar regions is elevated in AD patients, but its relationship with cognitive decline requires further study. The hippocampal regions are specifically enriched in iron, and dysregulation of iron homeostasis in this region is strongly associated with Aβ deposition, tau hyperphosphorylation, neuronal loss, and neuroinflammation. Of these, the hippocampal cornu ammonis 1 (CA1) region and the dentate gyrus are the most dramatically affected in AD (Junceda et al., 2024). Clinical studies have shown (Mandal et al., 2023) that AD patients have significantly lower serum iron levels but significantly higher iron levels in the hippocampal region compared to healthy individuals and those with mild cognitive impairment. Elevated iron levels in hippocampal tissue are closely associated with oxidative stress and are a precursor to early Aβ plaque formation and abnormal tau phosphorylation (Zhou et al., 2024). Iron accumulation in these regions exacerbates oxidative stress and promotes pathological changes in Aβ and tau, leading to neuronal damage and death, and ultimately causing severe impairments in memory and learning ability (Figure 1).

## 3 Iron accumulation-mediated phosphorylation modifications affect AD

### 3.1 Iron accumulation induces abnormal phosphorylation of Tau

**FIGURE 2:**
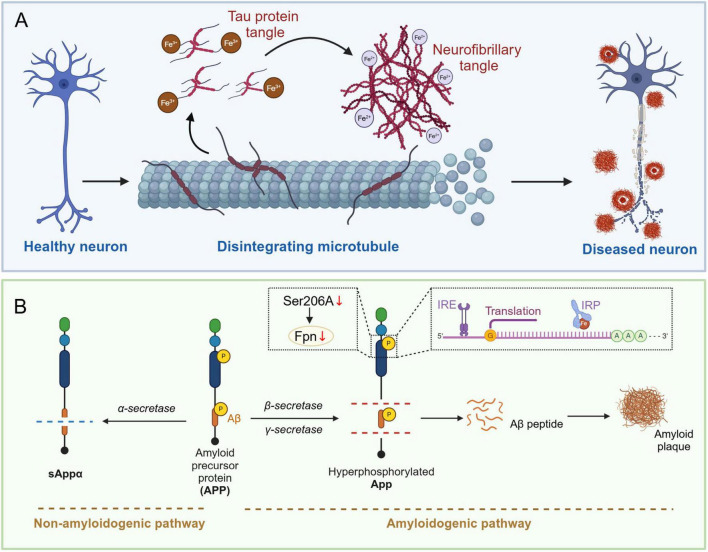
Iron-mediated Tau hyperphosphorylation and amyloid plaque deposition in the brains of Alzheimer’s disease patients. **(A)** Healthy neurons have intact microtubule structures and unphosphorylated Tau protein. When Fe^3 +^ (trivalent iron) accumulates, it promotes the aggregation of hyperphosphorylated Tau protein. When Fe^2 +^ (divalent iron) accumulates, it directly participates in the conformational change of Tau protein due to its reducibility, further promoting its aggregation. These changes ultimately lead to neuronal dysfunction and the formation of pathologically characteristic neurons. **(B)** APP (amyloid precursor protein) is metabolized along the non-amyloid pathway by alpha-secretase to produce sAPPα, whereas if it is processed by beta-secretase and gamma-secretase, it is metabolized along the amyloid pathway to produce the Aβ peptide, which is deposited to form amyloid plaques. In AD patients, phosphorylation of the Ser206 site on the iron regulatory protein (IRP) interacts with the iron response element (IRE), which in turn inhibits the expression of Fpn (iron transport protein) and promotes the production and deposition of Aβ peptides. APP, amyloid precursor protein; Aβ, amyloid beta; IRP, iron regulatory protein; IRE, iron-responsive element; FPN, ferroportin.

Tau is a microtubule-associated protein primarily responsible for stabilizing microtubule structures in neurons and is involved in intracellular transport (Pidara and Yamada, 2022). Microtubules are a crucial component of the cytoskeleton. Tau in their normally phosphorylated state ensure the stability and function of microtubules by regulating their binding capacity to microtubules (Barbier et al., 2019). Long axons and dendrites of neurons require robust scaffolds to maintain their morphology and function, and microtubule stabilization is particularly important for preserving neuronal structure and function (Kelliher et al., 2019). Furthermore, Tau are highly expressed in axons, and their phosphorylation status plays a regulatory role in the transport of substances within axons (Iwata et al., 2019). Axonal transport involves the directed movement of various molecules and organelles from the cell body to the synaptic terminal and is essential for normal neuronal function and long-range signaling (Guedes-Dias and Holzbaur, 2019). Normally phosphorylated Tau ensure smooth and efficient transport, thereby supporting neuronal growth, maintenance, and synaptic plasticity. By regulating the dynamic balance of microtubules and axonal transport, Tau indirectly affect the efficiency of synaptic transmission and the functional integration of neural networks, which are critical for higher functions such as learning and memory (Wang and Mandelkow, 2016). Conversely, in response to cellular stress and injury, the phosphorylation state of tau changes, and these changes may contribute to cellular adaptation and survival (Guo T. et al., 2017). The roles of phosphorylated tau in stress responses include regulating cytoskeletal reorganization, modulating cellular signaling pathways, and protecting cells from excessive damage. When neurons experience trauma or stress, dynamic phosphorylation of tau can facilitate rapid cellular responses and recovery, maintaining nervous system stability (Jenkins et al., 2000). Thus, by stabilizing microtubules, regulating axonal transport, maintaining neuronal signaling, and participating in cellular stress responses, phosphorylated tau ensure the health and function of the nervous system. The synergy of these activities is crucial for proper nervous system function and the realization of higher neural functions (Figure 2A).

In AD patients, tau phosphorylation primarily occurs at serine and threonine residues. Tau phosphorylation in AD has been reported to involve multiple sites, including Ser202/Thr205, Thr217, Thr212/Ser214, Thr181, Ser396, Ser422, Ser199, Thr231, and Ser262 (Mai et al., 2022; Hart de Ruyter et al., 2023; Hines et al., 2023). Hyperphosphorylation at these sites causes detachment from microtubules and leads to the formation of insoluble NFTs. This abnormal phosphorylation is a key pathological feature of AD. Mechanisms of tau phosphorylation include imbalances in kinases and phosphatases, oxidative stress, iron accumulation, and inflammatory responses. Among these, iron accumulation plays a crucial role in the phosphorylation and aggregation of tau (Rao and Adlard, 2018). Iron, the most abundant metal ion in the brain, can directly bind to the iron-binding motif in tau. Specifically, Fe^3 +^ binding to Tau induces the hyperphosphorylation of Tau, which promotes its aggregation into paired helical filaments (PHF Tau), ultimately contributing to the formation of NFTs (Yamamoto et al., 2002). The formation of NFTs can hinder the extracellular iron efflux pathway regulated by Tau, further exacerbating the accumulation of iron. However, this effect only occurs when iron is in its oxidized state (Fe^3 +^) and not when it is in its reduced state (Fe^2 +^). The redox state transition of iron plays a crucial role in the aggregation of PHF Tau. Studies indicate that Fe^3 +^ can promote the aggregation of Tau, while Fe^2 +^ affects the aggregation state by inducing reversible conformational changes in Tau and influences the formation of NFTs through interaction with Thr residues under the influence of Tau phosphorylation (Rao and Adlard, 2018). Further research has shown (Wan et al., 2019) that in primary neuronal cultures from sub-iron-treated SD rats and in C57BL/6 mice fed a high-iron diet, the phosphorylation levels of Tau at the Ser202/Thr205, Thr181, and Ser396 sites increased significantly, while total Tau expression remained unchanged, further explaining the pathological mechanism between iron and PHF tau.

### 3.2 Iron accumulation mediates abnormal phosphorylation of APP

Amyloid precursor protein is a transmembrane glycoprotein widely found in mammalian cell membranes, with particularly high expression in neurons. There are three main isoforms of APP, which contain 695, 751, and 770 amino acids, respectively. These isoforms are present in the brain and are generated by alternative transcriptional splicing (Hefter et al., 2020). APP can be processed through two main pathways: the non-amyloid pathway and the amyloid pathway. In the non-amyloid pathway, APP is cleaved by α-secretase (ADAM10) at amino acid position 17 of the Aβ sequence, generating sAPPα and C83 fragments. Subsequently, γ-secretase cleaves the C83 fragment to generate the P3 peptide and the APP intracellular domain, yielding a fragment that is protective for neuronal cells (Cho et al., 2022). In AD, however, phosphorylation of APP alters its metabolic pathway, making it more susceptible to cleavage by β- and γ-secretase, leading to the production of Aβ. In this process, phosphorylation occurs mainly in the intracellular segment of APP, such as at the threonine (Thr688/181) site, which has been shown to increase APP’s affinity for β- and γ-secretase, facilitating the production of Aβ and the formation of insoluble amyloid plaques (Lewczuk et al., 2020; Figure 2B).

Iron accumulation has been reported to affect iron storage and distribution by disrupting the interaction between iron-regulatory proteins IRP1, IRP2, and the iron-responsive element (IRE) (Khan, 2020). Further studies showed that the 5’ untranslated region of APP mRNA contains an IRE that binds iron-regulatory proteins. Iron can bind directly to IRP, leading to its dissociation from IRE, thereby promoting APP mRNA translation and Aβ production. Binding of IRP1 and IRP2 directly enhances APP translation (Zhou and Tan, 2017). Therefore, the degree of iron accumulation directly affects APP expression and Aβ production. More importantly, APP on the cell surface not only contributes to amyloid plaque formation but also maintains iron efflux by stabilizing the ferroportin (FPN), thus playing a key role in neuronal iron homeostasis. It has been shown (Tsatsanis et al., 2019) that phosphorylation of the extracellular region of APP (especially at site Ser206) is required for APP translocation to the cell surface, and that N-glycosylation and phosphorylation affect its localization and binding to FPN. Deletion of the phosphorylation site Ser206A resulted in a significant reduction in cell surface APP levels and a decrease in total soluble APP, suggesting that phosphorylation plays an important role in APP secretion. These mutations also led to a significant increase in intracellular iron levels, suggesting an important role of APP phosphorylation in iron efflux and intracellular iron regulation. Furthermore, using a neuroblastoma cell line stably expressing APP, it was found (Tsatsanis et al., 2019) that mutations in the phosphorylation and N-glycosylation sites not only reduced the presence of APP and FPN on the cell surface but also caused intracellular iron retention. This further revealed that these mutants exhibited significant iron accumulation in a high-iron environment, suggesting that APP plays an important role in regulating intracellular iron homeostasis. In addition, tau was found to further promote the transport of APP to the cell surface, aiding in iron export (Li et al., 2015). By injecting Aβ oligomers into the mouse hippocampus, it was found that eliminating tau prevented Aβ-induced cognitive impairment, neuronal loss, and iron accumulation. Increased APP levels, tau phosphorylation, and iron accumulation persisted even after Aβ was removed, suggesting that these factors continue to contribute to AD pathology. Additionally, transgenic mice expressing APP and progerin 1 (APP/PS1) treated with high doses of iron can serve as a model for AD. High-dose iron increases APP expression and phosphorylation, enhances amyloid APP cleavage and Aβ deposition, and impairs spatial learning and memory (Kupershmidt et al., 2012; Guo et al., 2013b). In contrast, the iron chelators deferoxamine and M30 can significantly improve memory in mice by inhibiting iron-induced amyloid APP processing, shifting APP processing to the non-amyloid pathway, reducing APP protein expression and phosphorylation at the Thr668 site, and attenuating Aβ deposition (Amit et al., 2017; Stergiou et al., 2023).

## 4 Iron accumulation-induced oxidative stress mediates aberrant phosphorylation of AD

### 4.1 Iron-mediated oxidative stress promotes hyperphosphorylation of Tau

Oxidative stress is a process in which excessive levels of ROS exceed the capacity of the body’s antioxidant defense mechanisms, leading to cellular damage. Although the pathological features of AD are dominated by amyloid Aβ plaques and hyperphosphorylation of tau, oxidative stress is an early event, occurring even before the formation of Aβ plaques and tau phosphorylation (Mandal et al., 2023). Excessive iron accumulation during AD pathology generates ROS via the Fenton reaction, producing highly reactive hydroxyl radicals that exacerbate oxidative stress and damage neurons and cellular structures. In an oxidative stress environment, the production of ROS exceeds the cell’s antioxidant capacity, making tau more prone to phosphorylation and aggregation. Previous studies have shown (Xie et al., 2012) that levels of the antioxidant glutathione in the hippocampus and plasma are significantly decreased in patients with AD, and that this reduction further exacerbates iron-mediated oxidative stress by lowering the cell’s antioxidant capacity. Treatment with the antioxidant ebselen was able to reverse metallotransporter protein 1-mediated iron transport and increase glutathione level (Xie et al., 2012). In addition, the initiation of signals such as cystathionine depletion and lipid peroxidation, associated with ferroptosis (iron-dependent cell death), leads to increased iron levels, making neurons more susceptible to ferroptosis, resulting in neuronal death (Lane et al., 2021). Related studies have shown (Li et al., 2022) that increased phosphorylation of nuclear factor erythroid 2-related factor 2 (Nrf2) at Ser40 promotes the release of Nrf2 from Kelch-like ECH-associated protein 1 and its translocation into the nucleus, which activates heme oxygenase-1 (HO-1) expression. This effectively reduced ferroptosis in the brains of APP/PS1 mice, thereby inhibiting Aβ-induced tau hyperphosphorylation and neurotoxic 1-42 oligomerization in HT-22 cells. However, another study found (Hui et al., 2011) that long-term overexpression of HO-1 significantly increased the release of iron from the catabolism of hemoglobin, enhanced the local iron load in the brain, and induced phosphorylation of tau at the Ser199/202/396 site, promoting the aggregation of tau in HO-1 overexpressing transgenic mice. The use of HO-1 inhibitors can reduce iron release and local iron accumulation, thereby reducing oxidative stress and inflammatory responses, and protecting neuronal function. Additionally, knockdown or pharmacological inhibition of HO-1 reduces iron accumulation and neuronal damage, and improves cognitive function in AD mouse models (Li et al., 2022). Thus, short-term or moderate activation of HO-1, an inducible enzyme that catabolizes heme to produce iron, carbon monoxide, and biliverdin/bilirubin, may be protective by reducing ferroptosis and lowering oxidative stress, whereas long-term overactivation increases iron release and accumulation, promoting neurotoxicity and tau phosphorylation.

### 4.2 Iron-mediated oxidative stress triggers dephosphorylation of apoptosis-related proteins

In addition to iron-mediated pro-phosphorylation, dephosphorylation also plays an important role in the pathogenesis of AD. BCL-xL/BCL-2-associated death promoter (BAD) is a pro-apoptotic protein in the Bcl-2 family, whose activity is regulated by its phosphorylation status. Unphosphorylated BAD can bind to anti-apoptotic proteins and block their inhibitory effects on pro-apoptotic factors, leading to cell death (Deng, 2017). In contrast, when BAD is phosphorylated by specific kinases (e.g., AKT/ Protein Kinase A), it binds to 14-3-3 proteins, which are then released from the mitochondria, losing their inhibitory effect on Bcl-2 family members (Fu et al., 2000). In AD, oxidative stress and cellular stress responses lead to BAD dephosphorylation, restoring its pro-apoptotic function and promoting neuronal apoptosis. Dephosphorylated BAD binds to Bcl-2 and Bcl-xL, inhibiting their anti-apoptotic functions, leading to loss of mitochondrial membrane potential and cytochrome c release, initiating the apoptotic cascade (Zhao et al., 2021). Studies have shown (Kuperstein and Yavin, 2003) that the combined action of Aβ and Fe^2 +^ significantly reduces protein kinase C (PKC) isoforms, decreases Akt kinase activity, and reduces BAD phosphorylation, while enhancing p38 MAPK and caspase-9 and -3 activation, exhibiting pro-apoptotic features. Iron chelators can effectively block these pro-apoptotic activities. Notably, although Aβ alone enhanced PKC isoforms, Akt activity, and BAD phosphorylation, and Fe^2 +^ could transiently enhance p38 MAPK and caspase activity, neither was sufficient to cause cell death alone. However, direct inhibition of the PKC or Akt pathway enhances Aβ/Fe^2 +^ toxicity, whereas inhibition of p38 MAPK prevents cell injury and apoptosis (Kuperstein and Yavin, 2003).

In addition, iron plays an important role in the pathogenesis of AD by regulating the phosphorylation of the Ser637 site of Dynamin-related protein 1 (Drp1), which consists of several structural domains, including the GTPase domain, the intermediate domain, and the GTPase Effector Domain (GED) (Park et al., 2015; Kandimalla et al., 2016). The GTPase domain is responsible for hydrolyzing GTP to provide energy, while the intermediate and GED domains are involved in protein interactions and multimerization (Hill et al., 2017). Drp1 exists as a monomer in the cytoplasm and aggregates at the outer mitochondrial membrane during mitochondrial division, forming a loop or helix by interacting with receptor proteins (e.g., Fis1, Mff, Mid49, Mid51) at the outer mitochondrial membrane. By interacting with these receptor proteins, Drp1 forms a ring or helical multimeric structure, splitting the mitochondria using energy generated from GTP hydrolysis (Osellame et al., 2016). In addition, by regulating mitochondrial fission and fusion, Drp1 plays a key role in maintaining mitochondrial quality and function, helping cells remove damaged or malfunctioning mitochondria (Zerihun et al., 2023). The pathogenesis of AD is often accompanied by cellular stress and injury, during which Drp1 is activated and translocated to the mitochondria to promote mitochondrial fission. This fission is often accompanied by a loss of mitochondrial membrane potential and increased permeability of the outer mitochondrial membrane, leading to the release of apoptotic factors such as cytochrome c, initiating a cascade of apoptotic responses (Kim et al., 2016). Studies have shown (Xie et al., 2012; Zhang et al., 2020) that excessive iron accumulation leads to dephosphorylation of Drp1 at Ser637, increasing the activity of dephosphorylated Drp1 and inducing excessive mitochondrial fragmentation and dysfunction. In contrast, inhibiting calmodulin phosphatase activation prevents iron-induced, Drp1-dependent mitochondrial fragmentation and apoptosis, maintains the phosphorylated state of Drp1 at Ser637, and protects neurons from iron overload (Park et al., 2015; Yu et al., 2022).

## 5 Iron-mediated phosphorylation of neurotransmitter receptors

### 5.1 NMDAR phosphorylation

The NMDAR is a key glutamate receptor and ion channel protein in the central nervous system. It is composed of three subunits: GluN1, GluN2, and GluN3, and plays a crucial role in synaptic transmission, synaptic plasticity, learning, memory, and other processes related to neuronal excitability (Yu et al., 2021). Under normal conditions, NMDARs influence calcium ion signaling by regulating synaptic strengthening and weakening mechanisms, gene expression, and neuronal survival and apoptosis (Zhang et al., 2007; Chen et al., 2014). Additionally, during neurodevelopment, NMDAR activation promotes the stabilization and refinement of dendritic spines, thus facilitating the formation of mature neural networks (Kehoe et al., 2014). It is noteworthy that NMDAR function is influenced by several factors, including magnesium blockade, phosphorylation, internalization and exocytosis, and regulatory proteins (Liu et al., 2023). Phosphorylation typically enhances receptor function, increases ion permeability, and regulates its localization on the cell membrane (Wang et al., 2014). In AD, abnormally phosphorylated tau can bind to the GK domain of PSD-95 through multivalent interactions, causing the dynamic arrest of PSD condensates and clusters, thereby affecting the dynamics and phosphorylation status of NMDA receptors. Specifically, this interaction can disrupt the tyrosine phosphorylation of the GluN2B subunit, leading to altered receptor trafficking and reduced synaptic stability. These changes further trigger calcium overload and neuroexcitotoxicity, even at normal glutamate concentrations. Moreover, persistent NMDAR overactivation, particularly involving GluN2B-containing extrasynaptic receptors, exacerbates chronic excitotoxicity, resulting in neuronal loss, dysfunction, and a decline in neural network function (Xuan et al., 2013; Miyamoto et al., 2017; Thakral et al., 2023). Additionally, NMDAR activation accelerates divalent metal transporter 1 (DMT1) mediated iron influx, with Fe^2 +^ and Ca^2 +^ entering the NMDAR channel competitively, promoting iron release from lysosomes, increasing intracellular iron levels in primary neurons, and exacerbating neuronal damage (Xu et al., 2017). Notably, a stable Fe^2 +^ level in neurons can generate ROS, activating NMDAR, increasing NMDAR-mediated Ca^2 +^, and further promoting Ca^2 +^ generation and persistence through Ryanodine receptor-mediated calcium release, maintaining normal intracellular redox homeostasis (Sanmartín et al., 2014). Previous studies have shown (Muñoz et al., 2011) that iron deficiency or the use of iron chelating agents can significantly reduce NMDAR-mediated calcium signaling and weaken intracellular calcium signaling. This reduction in calcium signaling and NMDAR-dependent extracellular signal-regulated kinases 1 and 2 (ERK1/2) phosphorylation leads to reduced synaptic transmission in the CA1 region of hippocampal neurons, impairing long-term potentiation (LTP) and, consequently, affecting learning and memory functions.

### 5.2 GABA_A_ receptors phosphorylation

The gamma-aminobutyric acid type A receptor (GABA_*A*_R) is one of the most important inhibitory neurotransmitter receptors in the central nervous system and is responsible for mediating fast inhibitory synaptic transmission in the brain (Ghit et al., 2021). The GABA_*A*_R is a ligand-gated chloride channel. When GABA binds to the receptor, the receptor channel opens, allowing chloride ions (Cl^–^) to enter the cell. The proper functioning of the receptor directly affects the hyperpolarization of the neuronal membrane and is essential for maintaining the excitatory-inhibitory balance of neurons (Sallard et al., 2021). Phosphorylation is an important mechanism for regulating GABA_*A*_R function in this process. By altering the structure, activity, and localization of the receptor, phosphorylation can rapidly and dynamically regulate inhibitory signal transmission (Saliba et al., 2012). Phosphorylation of GABA_*A*_R typically occurs in the intracellular region and primarily involves specific serine (Ser359, Ser343, Ser383, Ser327) and threonine (Thr375) residues (Kellenberger et al., 1992; Meier and Grantyn, 2004; Houston et al., 2007; Mukherjee et al., 2011; Nakamura et al., 2020). In physiological processes, the phosphorylation of GABA_*A*_R receptors is a key mechanism for regulating synaptic plasticity. The phosphorylation state of GABA_*A*_R can regulate the response of neurons to inhibitory signals, affecting learning and memory processes (Nakamura et al., 2015). Under stress conditions, the phosphorylation state of GABA_*A*_R changes, affecting neuronal excitability and the overall inhibitory tone of the brain, thereby regulating behavior and emotional responses (Maguire, 2014).

Today, benzodiazepines (BZDs) are widely used to treat anxiety, insomnia, seizures, and as anticonvulsants by binding with high affinity to the GABAAR and potentiating the effects of GABA (Furukawa et al., 2017). However, clinical reports suggest (Ettcheto et al., 2019) that long-term use of BZDs may increase the risk of AD. Additionally, chronic BZD intervention induces the overexpression and phosphorylation of the GABA_*A*_R subunit α5 in the mouse brain and significantly upregulates the expression of lipocalin 2 (Lcn2), resulting in intracellular iron overload, increased oxidative stress levels, and cell damage or death (Furukawa et al., 2017). Additionally, knocking down Lcn2 expression in hippocampal neurons from AD mice has been shown to alleviate glial activation, memory impairment, and synaptic abnormalities in mice (Zhou et al., 2023). Diazepam administration has also been found to increase Lcn2 expression in the cerebral cortex, hippocampus, and amygdala, a process that is not accompanied by the upregulation of pro-inflammatory genes and can be reversed by the iron chelator deferoxamine mesylate (DFO) (Furukawa et al., 2017). This suggests that the phosphorylation of GABA_*A*_R and the role of Lcn2 in the nervous system are closely related to the regulation of iron homeostasis. However, it is important to note that Lcn2, as an iron-related protein, plays a dual role in regulating intracellular iron homeostasis by binding to iron carriers. On one hand, Lcn2 binds to iron chelates, reducing the availability of free iron in the cell and thus preventing oxidative stress caused by iron overload (Devireddy et al., 2005); On the other hand, Lcn2 ensures that cells can obtain sufficient iron when needed by promoting the storage and utilization of iron ions (Xu et al., 2012). Therefore, changes in Lcn2 expression may be important in the regulation of iron homeostasis. Overall, although targeting Lcn2 expression by regulating the expression and phosphorylation state of GABA_*A*_R may offer a means to regulate iron metabolism, the potential benefits need to be carefully weighed against the risk of neurotoxicity. In the central nervous system, the complex regulatory relationship between GABA_*A*_R and Lcn2 requires careful consideration to ensure the safety and efficacy of long-term treatment.

## 6 Iron-mediated phosphorylation of tyrosine hydroxylase

Tyrosine hydroxylase (TH) is the rate-limiting enzyme in dopamine synthesis. It catalyzes the conversion of L-tyrosine to L-DOPA, which is subsequently decarboxylated to dopamine. TH is mainly found in dopaminergic neurons in the central nervous system, especially in the substantia nigra, striatum, and locus coeruleus (Depboylu et al., 2014; Dunkley and Dickson, 2019). TH expression and activity are essential for the normal function of dopaminergic neurons (Niu et al., 2022). TH function and activity are controlled by multiple regulatory mechanisms, with phosphorylation being one of the most important. TH is typically phosphorylated at Ser8, Ser19, Ser31, Ser40, and Thr8 (Niu et al., 2022). Additionally, TH can be regulated by various kinases, including PKA, PKC, and calcium/calmodulin-dependent protein kinase II (CaMKII). These kinases respond to changes in intracellular signals and regulate the activity of TH through phosphorylation, thereby affecting dopamine synthesis (Perfume et al., 2007; Yabuki et al., 2014).

Tyrosine hydroxylase is a tetramer, with each subunit approximately 60 kDa, containing a catalytic domain, a regulatory domain, and an N-terminal domain with multiple phosphorylation sites (Lewis et al., 1993). Since each subunit contains an iron ion, TH activity is dependent on iron as a cofactor (Xiao et al., 2021). Therefore, an imbalance in iron homeostasis can affect TH function. Excessive iron accumulation has been reported to catalyze the production of hydrogen peroxide, a by-product of dopamine metabolism, leading to oxidative stress, lipid peroxidation, and changes in membrane fluidity. These effects regulate kinase activity, inhibit the phosphorylation of TH, contribute to dopaminergic neuron dysfunction, exacerbate dopaminergic neuron death, promote pathological changes in Aβ and Tau, and ultimately worsen cognitive decline (Dev et al., 2015). Studies have shown (Farr and Xiong, 2021) that intranasal DFO treatment in an animal model of AD can significantly increase the survival rate of dopaminergic neurons and reduce iron-induced loss of TH-positive neurons, thereby maintaining normal dopamine synthesis. Additionally, the iron chelator cloxacillin can bind to excess free iron, reduce brain iron levels, increase the phosphorylation of tyrosine hydroxylase at Ser40 in the substantia nigra, reverse motor and cognitive deficits, and increase brain-derived neurotrophic factor (BDNF) levels in the hippocampus of Tau knockout mice (Lei et al., 2015).

## 7 Ca^2+^ and Tf phosphorylation

Transferrin is a glycoprotein responsible for iron transport. By binding to TfR1 or TfR2 on the cell surface, Tf delivers iron into the cell. This process ensures that cells obtain sufficient iron to meet their metabolic needs while preventing oxidative stress and cell damage caused by the accumulation of free iron ions (Ponka and Lok, 1999). Therefore, the phosphorylation of Tf may alter its structure, affecting its ability to bind to TfR and thereby regulating iron transport efficiency. Changes in the phosphorylation state of Tf may result in poor receptor binding, disrupting iron homeostasis and metabolism (Ma et al., 2023). In the central nervous system, the phosphorylation state of Tf directly affects iron uptake by neurons and the synthesis of neurotransmitters. Clinical data have shown that an abnormal increase in the phosphorylation level of Tf in the brains of AD patients is associated with decreased iron-binding capacity, leading to an increase in free iron levels in the brain, the production of large amounts of ROS, and the onset of oxidative stress (Ashraf et al., 2020). Additionally, abnormal Tf phosphorylation in cerebrospinal fluid and serum samples from both early (< 65 years) and late (> 65 years) AD patients has been associated with both early and late stages of the disease (Sabbir, 2018).

The Ca^2 +^-mediated calmodulin-dependent protein kinase kinase 2 (CaMKK2) signaling pathway is a multifunctional network involving calcium regulation, calmodulin-mediated kinase activation, and downstream signal transduction (Tokumitsu and Sakagami, 2022). Ca^2 +^ signals are typically triggered by extracellular stimuli, such as neurotransmitters, hormones, or other signals. External stimuli activate receptors such as NMDAR, leading to a rapid increase in intracellular Ca^2 +^ concentration (Ma et al., 2024). When intracellular Ca^2 +^ concentration rises, calcium ions bind to calmodulin and activate it. Each calmodulin molecule can bind four Ca^2 +^ ions, and this binding triggers a conformational change in calmodulin, allowing it to interact with downstream target proteins. The activated calmodulin binds to CaMKK2, inducing a conformational change and activating its downstream kinase activity, such as calcium/calmodulin-dependent protein kinase IV (CaMK4) (Najar et al., 2021). Since the Ca^2 +^/CaMKK2 signaling pathway plays a key role in neuronal survival and synaptic plasticity, numerous studies have demonstrated that dysregulation of Ca^2 +^, CaMKK2, and its upstream kinases (such as GSK3, CDK5, and PKA) induces AD symptoms (Marcelo et al., 2016). Previous studies have found that Ca^2 +^/CaMKK2 can affect the modification and transport of Tf through phosphorylation (Sabbir, 2018). Deletion of CaMKK2 leads to decreased phosphorylation of Tf at multiple functionally related residues (Ser/Thr/Tyr) and affects its acidic portion (pH 3–4). In CaMKK2 knockout mice and triple transgenic AD mouse models, Tf phosphorylation in the brain and serum is significantly reduce (Sabbir, 2018). Further studies have found that CaMKK2-mediated CaMK4 deletion leads to a significant increase in Tf phosphorylation (Ser/Thr/Tyr) and the expression of TfR in the mouse cerebellum (Sabbir, 2020). In contrast, the use of Ca^2 +^ chelators or Ca^2 +^ channel inhibitors can significantly reduce TfR circulation or accelerate Tf recycling (Sabbir, 2020). Therefore, abnormal Ca^2 +^ expression and downstream kinase-regulated Tf phosphorylation in AD, especially the regulation of iron homeostasis via tissue-specific phosphorylation of Tf and TfRc, provide new insights into how the CaMKK2-CaMK4 signal affects iron metabolism and highlight mechanisms that require further exploration (Table 1).

**TABLE 1:** Iron metabolism mediates protein phosphorylation in Alzheimer’s disease (AD) pathogenesis.

	Type	Intervention	Phosphorylation site	Gene expression relative to controls	Mechanism of action	References
Tau	Iron-overload model mice	High-dose iron rail feeding	Ser202/Thr205; Thr181; Ser396	Increased	• Fe^3 +^ reverses PHF tau aggregation • Release of soluble PHF tau and Fe^2 +^ • Promoting the formation of NFTs	Wan et al., 2019
	Rat primary neurons	High-dose iron rail feeding		Increased		
	Mouse hippocampal neuronal cells	Aβ-induced	p-Tau	Decreased	• Inhibition of Aβ-induced Tau hyperphosphorylation in Mouse hippocampal neuronal cells	Li et al., 2022
	Transgenic mice	HO-1 overexpressing	Ser199/202/396	Increased	• Enhanced iron loading and tau phosphorylation in the brain	Hui et al., 2011
APP	N2a neuroblastoma cell-lines	High-dose iron environments	S206A	Decreased	• Reduces APP Stability at the Cell Surface and Reduces FPN Levels, Leading to Iron Retention	Tsatsanis et al., 2019
	APP/PS1 double transgenic mice	High-dose iron rail feeding	Thr668	Decreased	• APP and Aβ deposition increased and impaired spatial learning and memory	Kupershmidt et al., 2012; Guo et al., 2013b
BAD	Neuronal cells	Aβ/Fe-induced	Ser136	Decreased	• Inhibition of Akt-dependent BAD phosphorylation, promoting apoptosis	Kuperstein and Yavin, 2003
Drp1	Mouse hippocampal neuronal cells	ferric ammonium citrate	Ser637	Decreased	• Iron overload will increase the activity of dephosphorylated Drp1, inducing mitochondrial fragmentation and dysfunction.	Park et al., 2015; Yu et al., 2022
NMDAR	Neuronal cells	Deferoxamine	p-ERK1/2	Decreased	• Iron Deficiency Reduces NMDAR-Mediated Calcium Signaling and ERK1/2 Phosphorylation Levels, Consistent with Synaptic Transmission in the CA1 Region	Muñoz et al., 2011
GABA_*A*_R	Mouse brain	Chronic BZDs	p-GABA_*A*_Rα5	Increased	• Up-regulation of Lcn2 expression results in intracellular iron overload, leading to cell injury or death	Furukawa et al., 2017
Tyrosine hydroxylase	Mouse hippocampus	Tau knockout	Ser40	Decreased	• Iron accumulation inhibits tyrosine hydroxylase phosphorylation and reduces BDNF expression, leading to cognitive and motor deficits, brain atrophy, and iron accumulation	Lei et al., 2015
Transferrin	Mouse	triple-transgenic mouse	Ser/Thr/Tyr on transferrin	Decreased	• Loss of CaMK4 leads to increased TfRc expression and altered transferrin phosphorylation	Sabbir, 2018

Tau, tubulin associated unit; NFTs, neurofibrillary tangles; PHF, paired helical filaments; HO-1, heme oxygenase-1; APP, amyloid precursor protein; FPN, ferroportin; AKT, protein kinase B; BAD, BCL2 associated agonist of cell death; Drp1, dynamin-related protein 1; Erk1/2, extracellular signal-regulated kinase 1 and 2; NMDAR, N-methyl-D-aspartate receptor; BZDs, benzodiazepines; GABA_*A*_R*_α_* 5, gamma-aminobutyric acid type A receptor α 5; CA1, cornu ammonis 1; Lcn2, lipocalin 2; BDNF, brain-derived neurotrophic factor; CaMK4, calcium/calmodulin-dependent protein kinase IV; TfRc, transferrin receptor; Ser, serine; Thr, threonine; Tyr, tyrosine.

## 8 Phosphorylation signaling network in the pathological mechanism of iron-mediated AD

Iron can affect the activity of many kinases and phosphatases, thereby regulating the phosphorylation state of key proteins. Phosphorylation signaling networks regulate many cellular functions through complex phosphorylation and dephosphorylation reactions. Their dynamic regulation and cross-interactions between pathways form the basis of the cell’s response to external stimuli and play an important role in the development and progression of many diseases. Many studies have shown (Kupershmidt et al., 2011; Roy et al., 2023) that abnormal iron metabolism in the body mediates the expression of various kinases such as GSK3β, CDK5, and MAPK, forming a complex phosphorylation network, increasing intracellular iron uptake and oxidative stress, promoting the hyperphosphorylation of Tau, which in turn induces abnormal aggregation of Tau, forming insoluble fibrillar aggregates, leading to loss of neuronal structure and function.

### 8.1 PI3K/AKT/GSK3β signaling pathway

In AD, abnormal phosphorylation of AKT and GSK3β leads to increased activity, which further promotes the overphosphorylation of Tau, forming NFTs, ultimately leading to the destruction of the structure and function of neurons (Zhao et al., 2024). In this process, hyperphosphorylated Tau can cause iron deposition, activate recombinant calcineurin (CaN) to dephosphorylate the Ser9 site of GSK3β, relieving the inhibition of GSK3β and leading to an increase in GSK3β activity, which in turn mediates the hyperphosphorylation of tau. At the same time, CaN further dephosphorylates the cyclic adenosine monophosphate response element binding protein, causing synaptic defects and memory impairment, creating a vicious cycle (Zuo et al., 2024). Previous studies have shown that in erastin (iron death inducer)-treated N2a cells (a cell line of mouse neuroblastoma), iron death inhibitors can significantly restore erastin-induced GSH depletion and GPX activity reduction, reduce ROS and iron levels, decrease GSK-3β activation (restore Ser9 phosphorylation levels), and ultimately reduce Tau hyperphosphorylation and aggregation (Wang et al., 2022). Zhang et al. (2015) found in an animal study that oral deferoxamine intervention can reduce iron accumulation induced by intraperitoneal injection of lipopolysaccharide (LPS) in the mouse brain by chelating iron ions, thereby reducing neuroinflammation and apoptosis. In addition, DFO further reduced LPS-induced neuroinflammation and cognitive impairment by increasing the phosphorylation of GSK3β at Ser9 and inhibiting its activity (Zhang et al., 2015). In addition to targeting iron accumulation, activation of vitamin D receptor agonists can also significantly reduce iron accumulation in the cerebral cortex and hippocampus of APP/PS1 mice by downregulating IRP2 and TfR, and reduce the phosphorylation of Tau at Ser396 and Thr181 sites by inhibiting the phosphorylation of GSK3β (Tyr216) (Wu et al., 2022).

In addition, GSK3β is active in the basal state and does not require activation by external signals. It can phosphorylate multiple substrates, including glycogen synthase, transcription factors, and other proteins. When growth factors (such as insulin, Insulin-like growth factor 1, and epidermal growth factor) bind to their corresponding receptor tyrosine kinases, the receptors self-phosphorylate and activate phosphoinositide 3-kinase (PI3K). PI3K phosphorylates the membrane phospholipid phosphatidylinositol 4,5-bisphosphate (PIP2) to generate phosphatidylinositol (3,4,5)-trisphosphate (PIP3) (Maqbool et al., 2016; Hermida et al., 2017). PIP3 acts as a second messenger to recruit AKT with a pleckstrin homology domain to the cell membrane. AKT is fully activated by phosphorylation at Thr308 by 3-phosphoinositide-dependent protein kinase 1 and at Ser473 by mechanistic target of rapamycin complex 2. The activated AKT inactivates GSK-3β by directly phosphorylating its Ser9 site. This phosphorylation alters the conformation of GSK-3β, preventing it from binding to substrates and reducing its kinase activity (Truebestein et al., 2021). In AD, iron accumulation and ROS produced by lipid peroxidation can oxidize the cysteine residues in the AKT molecule, forming disulfide bonds or sulfhydryl oxides, leading to a decrease in the phosphorylation level of AKT (Rochette et al., 2022). Studies have shown (Uranga et al., 2013) that exposing the mouse hippocampal neuronal cell line HT22 to 25–200 μM Fe2^+^ for 24 h activates the PI3K/AKT pathway, resulting in increased phosphorylation of AKT and GSK3β. It is worth noting that PI3K inhibitors and AKT inhibitors can increase iron-induced oxidative levels in HT22 cells, but GSK3β inhibitors have no significant effect, which further indicates the key role of AKT activity in inhibiting GSK3β and the production of oxidants (Uranga et al., 2013). Similarly, intervention with the iron chelator M30 in rat primary cortical cells directly enhanced the levels of phosphorylated AKT (Ser473) and phosphorylated GSK-3β (Ser9) and reduced the phosphorylation of Tau, protecting neurons from Aβ toxicity (Avramovich-Tirosh et al., 2010).

In addition, both iron accumulation and a high-fat diet can lead to cognitive deficits, and their combination can cause more severe cognitive damage. This combination also induces insulin resistance and alters insulin signaling pathways in the hippocampus, including reduced phosphorylation of AKT (do Nascimento et al., 2024). Normally, insulin binds and self-phosphorylates, leading to the activation of insulin receptor substrate 1 (IRS-1). Phosphorylated IRS-1 recruits and activates PI3K. The regulatory subunit of PI3K, PI3K p85α, binds to phosphorylated IRS-1 and activates PI3K, converting PIP2 to PIP3, which subsequently recruits and activates AKT (Kearney et al., 2021). Studies have shown (Wan et al., 2019) that ferrous chloride can reduce tyrosine phosphorylation levels of insulin receptor β (IRβ), IRS-1, and PI3K p85α in primary neurons, thereby disrupting the insulin signaling pathway and impairing learning and memory functions. Exogenous insulin supplementation can reverse the abnormal phosphorylation of tau induced by iron, restore tyrosine phosphorylation of IRβ, IRS-1, and PI3K p85α, and activate insulin signaling, thereby improving iron accumulation-induced neuronal pathology (Wan et al., 2019).

### 8.2 MAPK/P38/HIF-1 signaling pathway

The role of the MAPK/P38/HIF-1 regulatory network is crucial in the cellular response to external stress and hypoxic environments (Guan et al., 2020). Among these, hypoxia-inducible factor-1α (HIF-1α) is a transcription factor stabilized under hypoxic conditions, and its activity is regulated by phosphorylation (Daly, 2020). Under normal physiological conditions, cell surface receptors (e.g., Receptor Tyrosine Kinases, G Protein-Coupled Receptors) autophosphorylation and activate downstream rat sarcoma proteins after binding to their corresponding ligands (e.g., growth factors, cytokines). This activates rapidly accelerated fibrosarcoma kinase, which phosphorylates and activates MAPK/ERK kinase (MEK), which in turn phosphorylates and activates ERK (extracellular signal-regulated kinase) (Zhao and Luo, 2022). In AD, oxidative stress and the inflammatory response induced by iron accumulation activate upstream mitogen-activated protein kinase kinase 3/6 (MKK3/6), which phosphorylates and activates p38 MAPK. The activated p38 MAPK translocates to the nucleus, promoting the transcription and translation of HIF-1α (Fang et al., 2017). This phosphorylation stabilizes HIF-1α and promotes the expression of hypoxia-related genes. Meanwhile, under stress conditions, the p38 MAPK and HIF-1 pathways are cross-regulated to jointly respond to hypoxia and oxidative stress.

Studies have shown that aberrantly activated HIF-1α upregulates the expression of various downstream genes, including pro-inflammatory factors and vascular endothelial growth factor (VEGF), the overexpression of which exacerbates neuroinflammation, cerebrovascular lesions, and neuronal damage (Fehsel, 2023). Additionally, HIF-1α phosphorylation plays a key role in the pathogenesis of AD through multiple mechanisms, including promoting neuroinflammation, increasing Aβ accumulation, disrupting mitochondrial function, and accelerating neuronal death (Guo C. et al., 2017). The neuroprotective effects of iron chelators in AD have been shown to involve mechanisms such as reducing ROS production and stabilizing HIF-1α expression (Fine et al., 2012). *In vivo* studies have shown (Guo et al., 2015) that desferrioxamine reduces Aβ deposition and synaptic loss by upregulating HIF-1α mRNA and protein levels and inducing proteins encoded by HIF-1α adaptor genes, including TfR, DMT1, and BDNF. *In vitro* studies have shown (Guo et al., 2015) that DFO enhances the stability and phosphorylation levels of HIF-1α through activation of the P38/HIF-1α pathway, which further reduces iron in the CA3 region of the hippocampus and has no significant effect on the cortical and CA1 regions. Similarly, M30, a multifunctional iron chelator, was shown to increase HIF-1α protein levels in various brain regions and the spinal cord of adult mice, induce the expression of its dependent target genes such as VEGF, erythropoietin, TfR, HO-1, and other proteins, and further enhance the transcriptional levels of BDNF and growth-related protein-43 (Kupershmidt et al., 2011). These findings suggest that iron chelators activate the P38 MAPK signaling pathway to upregulate HIF-1α, increase the expression of HIF-1α-dependent genes, and participate in the pathological process of AD, with the mechanism potentially promoting the redistribution of iron rather than its removal for neuroprotective effects.

### 8.3 PKC/IRP1 signaling pathway

As mentioned earlier, IRP1 is a key protein responsible for regulating iron metabolism in mammalian cells. IRP1 regulates the stability and translation of iron metabolism-related mRNAs (such as APP) by binding to IREs, thereby influencing intracellular iron homeostasis. In general, IRP1 exhibits high IRE-binding activity, and this enhanced activity enables IRP1 to more effectively regulate mRNAs containing IREs, thereby modulating iron uptake and storage (Calvo-López et al., 2023). In this process, PKC, a serine/threonine kinase widely involved in cell signaling and regulation of various cellular functions, affects the phosphorylation state of IRP1. Specifically, PKC can phosphorylate IRP1 at the Ser-138 site, increasing its IRE-binding activity (Brown et al., 1998; Johnson et al., 2017). Additionally, the phosphorylation state of PKC affects the stability of the iron-sulfur (Fe-S) cluster of IRP1, making it more susceptible to oxidative stress. This, in turn, alters its dissociation from cytosolic aconitase to an IRE-binding protein, indirectly affecting the expression of TfR and ferritin (Brown et al., 1998). Several studies have shown (Alkon et al., 2007; Lu et al., 2022) that PKC phosphorylation levels are closely related to AD, and that abnormal Aβ accumulation and stress can regulate PKC activity. In addition, PKC overactivation can affect APP cleavage and rebalance the APP production pathway. Furthermore, the levels of PKC isoenzymes in nerve cells treated with Aβ and Fe^2 +^ were significantly increased. Both Ca^2 +^-dependent and non-dependent isoenzymes showed a significant increase within 4 h, while PKC levels significantly decreased after 12 h (Kuperstein and Yavin, 2003). This may indicate that prolonged exposure to Aβ and Fe^2 +^ leads to PKC depletion or negative feedback regulation. Further studies have shown that treating cortical primary cells from adult rats with the iron chelator M30 enhances PKC phosphorylation and upregulates neuroprotective adaptive mechanisms in the brain.

## 9 Conclusion and prospects

The pathological mechanisms of AD are complex and multifaceted, with protein phosphorylation processes mediated by the iron metabolism regulatory network playing a critical role in AD progression. Iron accumulation significantly influences the phosphorylation status of key proteins by directly binding to Tau and amyloid APP, inducing oxidative stress, and regulating the activity of various kinases and phosphatases. This, in turn, promotes the formation of NFTs and amyloid plaques. Additionally, phosphorylation is involved not only in the metabolic regulation of Tau and Aβ but also in multiple physiological processes, including neuroinflammation, synaptic function, and neuronal apoptosis. Targeting iron metabolism-related phosphorylation not only directly regulates iron homeostasis but may also indirectly influence the balance between iron metabolism and other cellular functions, such as synaptic and mitochondrial activity. This comprehensive approach could enhance neuronal health and intervene in disease progression at multiple levels. Although the role of iron in AD has garnered significant attention, the precise distribution and dynamic changes of iron at various subcellular levels have yet to be fully elucidated. Furthermore, the interaction between iron and other metal ions, such as copper and zinc, and their synergistic or antagonistic effects in phosphorylation regulation, require further exploration. Moreover, existing animal models and *in vitro* experimental systems cannot fully replicate the pathological characteristics and complex environment of human AD, leading to uncertainty regarding the applicability and feasibility of some research findings in clinical settings. Importantly, although iron chelators can slow AD progression by removing excess iron from the brain, their clinical application faces several challenges. Most iron chelators (e.g., deferoxamine, deferiprone, deferasirox) are unable to effectively penetrate the BBB, limiting their capacity to directly address iron accumulation in the brain (Roy et al., 2010; Farr and Xiong, 2021). Furthermore, iron chelators can affect iron metabolism throughout the body, potentially leading to side effects such as anemia, liver damage, and kidney dysfunction, which limit the safety of long-term use (Sadia et al., 2022). Additionally, although some preclinical and small-scale clinical trials have shown that iron chelators can improve memory and reduce pathological markers, clinical trial results have been inconsistent (Crapper McLachlan et al., 1991; Hatcher et al., 2009; Mohamed et al., 2019).

Therefore, future research should aim to provide a deeper understanding of the mechanisms underlying iron-mediated phosphorylation regulation, particularly regarding cellular specificity and temporal dynamics. Additionally, exploring the complex relationships between iron and other metal ions, as well as their regulatory networks, will aid in fully elucidating the pathological processes of AD. By selectively inhibiting or activating specific kinases or phosphatases that phosphorylate key proteins associated with AD, it may be possible to regulate molecules critical to the disease process without affecting normal cell function. Optimizing therapeutic strategies that target the pathological changes mediated by the iron metabolism regulatory network—including novel iron chelating agents, innovative drug delivery systems across the BBB, and kinase/phosphatase regulators—coupled with precision medicine approaches, will help further evaluate the long-term safety and efficacy of these agents and other potential treatments, ultimately aiming to significantly improve the prognosis of AD patients (Liu et al., 2010).
